# Non-Invasive Venous waveform Analysis (NIVA) for volume assessment during complex cranial vault reconstruction: A proof-of-concept study in children

**DOI:** 10.1371/journal.pone.0235933

**Published:** 2020-07-08

**Authors:** Jenna H. Sobey, Srijaya K. Reddy, Kyle M. Hocking, Monica E. Polcz, Christy M. Guth, Cameron Schlegel, Jon Whitfield, Susan S. Eagle, Colleen M. Brophy, Bret D. Alvis

**Affiliations:** 1 Department of Anesthesiology, Division of Pediatric Anesthesiology, Monroe Carell Jr. Children’s Hospital at Vanderbilt University Medical Center / Vanderbilt University School of Medicine, Nashville, Tennessee, United States of America; 2 Department of Surgery, Vanderbilt University Medical Center / Vanderbilt University School of Medicine, Nashville, Tennessee, United States of America; 3 VoluMetrix, LLC, Nashville, TN, United States of America; 4 Department of Anesthesiology, Division of Cardiothoracic Anesthesiology, Vanderbilt University Medical Center / Vanderbilt University School of Medicine, Nashville, Tennessee, United States of America; 5 Department of Surgery, Division of Vascular Surgery, Vanderbilt University Medical Center / Vanderbilt University School of Medicine, Nashville, Tennessee, United States of America; 6 Department of Anesthesiology, Division of Critical Care Medicine, Vanderbilt University Medical Center / Vanderbilt University School of Medicine, Nashville, Tennessee, United States of America; University of Alberta, CANADA

## Abstract

**Background:**

Non-Invasive Venous waveform Analysis (NIVA) is novel technology that captures and analyzes changes in venous waveforms from a piezoelectric sensor on the wrist for hemodynamic volume assessment. Complex cranial vault reconstruction is performed in children with craniosynostosis and is associated with extensive blood loss, potential life-threatening risks, and significant morbidity. In this preliminary study, we hypothesized that NIVA will provide a reliable, non-invasive, quantitative assessment of intravascular volume changes in children undergoing complex cranial vault reconstruction.

**Objective:**

To present proof-of-concept results of a novel technology in the pediatric population.

**Methods:**

The NIVA prototype was placed on each subject’s wrist, and venous waveforms were collected intraoperatively. Estimated blood loss and fluid/blood product administration were recorded in real time. Venous waveforms were analyzed into a NIVA value and then correlated, along with mean arterial pressure (MAP), to volume changes. Concordance was quantified to determine if the direction of change in volume was similar to the direction of change in MAP or change in NIVA.

**Results:**

Of 18 patients enrolled, 14 had usable venous waveforms, and there was a significant correlation between change in NIVA value and change in volume. Change in MAP did not correlate with change in volume. The concordance between change in MAP and change in volume was less than the concordance between change in NIVA and change in volume.

**Conclusion:**

NIVA values correlate more closely to intravascular volume changes in pediatric craniofacial patients than MAP. This initial study suggests that NIVA is a potential safe, reliable, non-invasive quantitative method of measuring intravascular volume changes for children undergoing surgery.

## Introduction

In pediatric patients, reliable determination of intravascular volume status can be challenging, as changes in vital signs often do not occur until significant blood loss and end-organ damage have ensued [1, 2]. Hemorrhage during surgery represents a major cause of pediatric perioperative morbidity, and early resuscitation improves survival [3, 4]. The Pediatric Perioperative Cardiac Arrest Registry reports that under-resuscitation of hemorrhage is one of the principal causes of perioperative death in children [5, 6].

Though there are some evolving predictors for determining volume responsiveness in adult patients, it is well known that hemodynamic parameters are poor predictors of fluid responsiveness in children [7–9]. Although it has not been shown to reliably reflect cardiac output [10, 11], mean arterial pressure (MAP) is still a widely utilized marker for volume assessment in children during anesthesia due to lack of other reliable predictors and the ability to obtain this measurement non-invasively. Thus, there is a large unmet need for developing and validating a method to accurately and non-invasively measure volume changes in the pediatric population.

Non-Invasive Venous waveform Analysis (NIVA) is novel technology that is designed to capture peripheral venous waveforms non-invasively through an innovative use of a piezoelectric sensor placed over the superficial veins of the wrist. The peripheral venous system is notably more compliant than the arterial and central venous compartments and serves as a volume reservoir, storing up to 70% of circulating blood volume. Therefore, any changes in blood volume are reflected first in the peripheral venous system. Because it is such a low-pressure system, it had not been extensively studied until now. With this innovative technology, the venous waveform is amplified and the lower frequencies that correspond to the pulse rate, and its harmonics (multiples of the pulse rate) are analyzed based on the ratiometric relationship of their respective amplitudes to hemodynamic volume changes. The result of said analysis is a NIVA value—an adjusted numerical value initially developed in adult patients undergoing elective right heart catheterization and considered to be “equivalent” to the clinical gold standard of volume status, pulmonary capillary wedge pressure [12, 13]. A recent study in adults showed that the NIVA value correlated linearly with blood loss and with the hemodynamic indices [14].

Complex cranial vault reconstruction (CCVR) is performed in infants and children with craniosynostosis to improve physical appearance, prevent functional neurological disturbances, and enhance psychosocial development. The complexities of the surgery can lead to extensive blood loss in patients with potential life-threatening risks and significant morbidity. Determining volume status and early detection of hemorrhage in this specific pediatric surgical population is problematic, and MAP, though not reliable, is often used clinically in CCVR as a marker to guide resuscitation. The aim of this pilot study was to provide proof-of-concept data in children that NIVA is a safe and non-invasive method for determining intravascular volume status. We hypothesized that NIVA values will more accurately correlate to volume changes compared to MAP in pediatric patients undergoing CCVR.

## Materials and methods

The University of Alabama Institutional Review Board (IRB) approved this study in a conflict of interest agreement with Vanderbilt University Medical Center IRB (#141848; Approval date: August 31, 2016). Written informed consent was obtained from all subjects, a legal surrogate, the parents or legal guardians for minor subjects. Patients who underwent craniofacial surgery at Monroe Carell Jr. Children’s Hospital at Vanderbilt between December 2016 and September 2018 were identified. From these, only patients who underwent CCVR, defined as fronto-orbital advancement/anterior cranial vault reconstruction, middle/posterior vault reconstruction, or total cranial vault reconstruction, were selected. Patient demographic data, including age, weight, race, ethnicity, American Society of Anesthesiologists (ASA) physical classification grade, and length of operative time were collected.

All patients underwent routine induction and maintenance of anesthesia. After intravenous access was obtained and the airway was secured, the NIVA prototype was secured to the volar aspect of each subject’s wrist using a Velcro® wristband, remaining in place for the duration of the surgery. Continuous, de-identified raw waveform data was recorded, amplified, and transmitted to a dedicated android tablet. The signal was then converted from the time domain to the frequency domain with a Fast Fourier Transformation (FFT), allowing identification of the various frequency components of the raw signal, correlating to the pulse rate and to higher harmonics, or multiples, of the pulse rate. These frequencies were identified with automated C++ software written to take 8192 sample windows, which then calculated the power of each cardiac frequency and utilized an algorithm that incorporated the relative power of each frequency into a NIVA value. NIVA values were recorded at 15-minute intervals from the software output files. Estimated blood loss (EBL) along with fluid/blood product administration was recorded in real-time. These values were used to assess changes in volume. Periods where signal quality was low were excluded from the analysis.

Changes in NIVA values and MAP, in relation to volume changes, were analyzed with Pearson correlation. To assess if the directional change in volume correlated to that of change in MAP or NIVA, percent concordance was quantified. GraphPad Prism 7 was utilized for statistical analysis. Concordance was calculated by determining a correct or incorrect change in NIVA or MAP respectively to EBL plus administrated fluid and a Cohen kappa was used to compare the effectiveness of the two measures.

## Results

**Fig 1:**
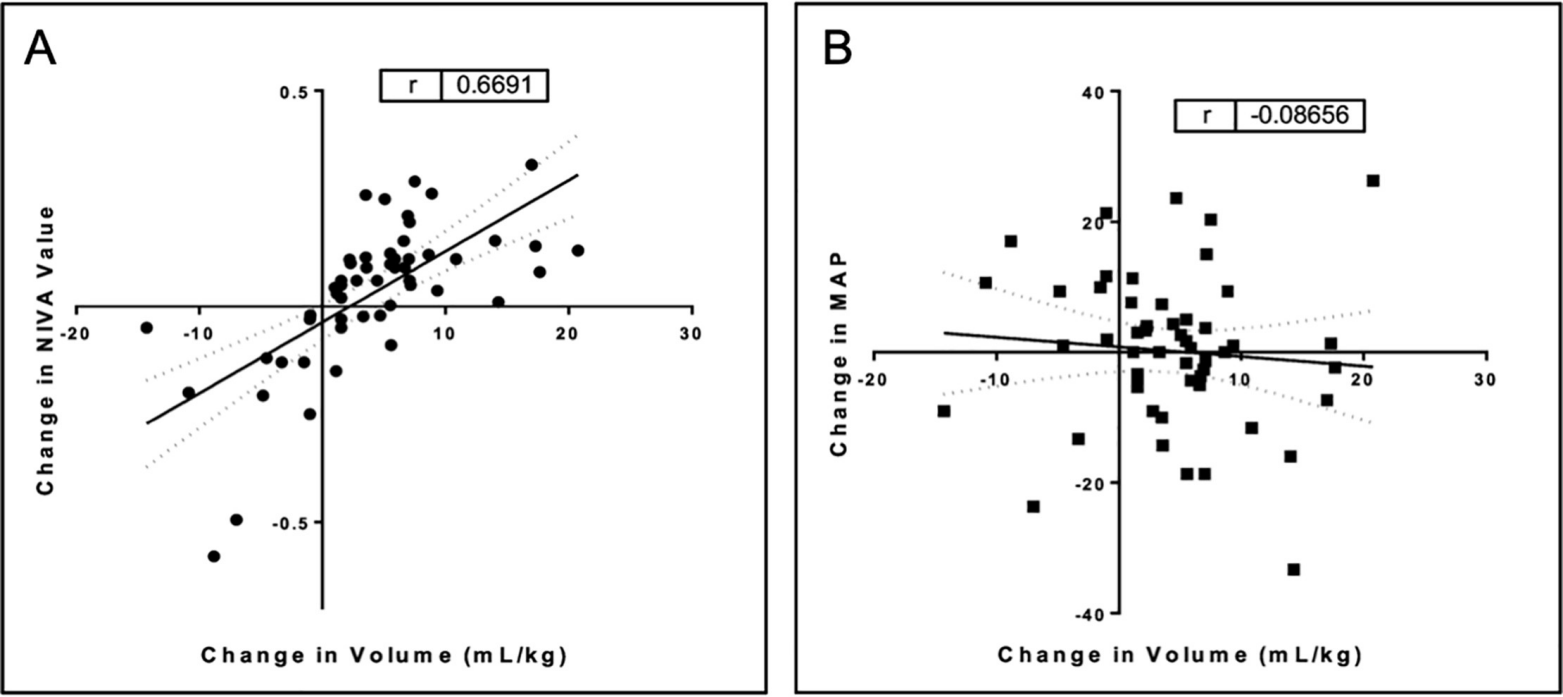
Change in volume (includes fluid/blood administration and estimated blood loss) vs. (A) change in NIVA signal and (B) change in MAP.

Eighteen children from 4 months to 8 years of age with craniosynostosis undergoing CCVR were enrolled. Venous waveforms were obtained from all 18 subjects. Four subjects were excluded from analysis due to systemic vasoactive agents affecting the signal. Demographics are presented in Table 1. There were no complications related to the use of the device. There was a significant correlation between the overall change in NIVA value with the net change in volume, defined as the sum of EBL and fluid/blood administration normalized to body weight, during CCVR procedures (r = 0.67, CI = 0.49 to 0.80, p < 0.05, Fig 1A). Meanwhile, change in MAP did not correlate with net change in volume (r = -0.09, CI = -0.35 to 0.19, p > 0.05, Fig 1B). The percent concordance between overall change in MAP and change in volume (54.7%) was less than the concordance between overall change in NIVA and change in volume (87.5%, p < 0.05). Using Cohen’s kappa model for determining the quality of agreement of NIVA when compared to MAP, a result of κ_2_ = 0.72 which is classified as a “good” quality of the agreement. The confidence intervals for Cohen’s kappa resulted in a range of 0.39 to 1.06.

**Table 1:** Demographics and other characteristics.

	N (%)	Mean ± SD	Median (Min-Max)
Number of Subjects	14 (100)		
Age (months)		23 ± 27	10 (4–84)
Weight (kg)		13 ± 8	9 (7–29)
Sex			
Female	5 (36)		
Male	9 (64)		
Race			
Asian	0 (0)		
Black	0 (0)		
Other	0 (0)		
White	14 (100)		
Ethnicity			
Hispanic	0 (0)		
Non-Hispanic	14 (100)		
ASA classification			
1	0 (0)		
2	12 (86)		
3	2 (14)		
Operative time (minutes)		190 ± 58	183 (115–329)

## Discussion

Determining volume status and detecting volume changes in pediatric patients while under anesthesia during major surgery is challenging. Clinical assessment of these fluctuating volume states based on exam and routine monitoring is inadequate, particularly in children [1, 4, 15]. It is often technically challenging or impractical to utilize invasive monitoring and unlike in the adult population, studies performed in children evaluating predictors of volume change have not consistently validated any current methods of monitoring [15–17]. The pediatric population poses several challenges with hemodynamic monitoring including a wide spectrum of ages, sizes, and weights. Additionally, vascular access in children, especially in neonates and infants, is often challenging.

A multitude of hemodynamic variables have been postulated to help monitor volume status and volume changes, including both static variables (heart rate, systolic blood pressure, central venous pressure, pulmonary capillary wedge pressure) and dynamic variables (systolic arterial pressure variation, pulse pressure variation, change in pulse oximeter plethysmography, plethysmography variability index, and stroke volume variation) [7–9, 16, 18, 19–21]. Most pediatric studies suggest that invasive strategies such as stroke volume variation (with a threshold value between 10 and 15%) with echocardiography and respiratory variation in aortic blood flow peak velocity with transesophageal echocardiography are the best predictors of volume status [7, 8, 16, 18, 22]. However, these approaches are not practical or possible in the intraoperative setting for most pediatric patients, due to the lack of accessibility and patient positioning during surgical procedures, particularly those occurring in the head and neck where a transthoracic echocardiogram cannot be placed. Results are mixed for plethysmography variability index, and it has not consistently been shown to predict volume status in pediatric patients [7, 8, 16]. Pulse pressure variation derived from automated waveform analysis still remains unproven as a reliable indicator of volume status in children [7, 8, 16, 18, 21]. This may be secondary to the increased arterial compliance that is noted in young children when compared to adults, particularly in those 0 to 2 years of age [23].

The NIVA prototype is unique in its ability to measure low-intensity venous waveforms *noninvasively* that have not previously been explored as a physiologic signal in children. As mentioned, the venous system is a highly compliant, low-pressure system that can accommodate large changes in volume with only minimal changes in pressure. Systemic venous compliance is higher than systemic arterial compliance, allowing the peripheral venous system to serve as a volume reservoir. Thus, changes in circulating blood volume have the greatest effect on venous blood volume.

In this pilot study, we were able to reliably obtain peripheral venous signals from pediatric patients undergoing CCVR, which were successfully amplified for analysis. These changes in waveform morphology that were analyzed at different volume states have demonstrated changes in the weighted power of the frequencies correlating to the pulse rate (f_0_) and the higher harmonics of the pulse rate (f_1-2_) relative to the total power of the cardiac component of the signal. When these changes are quantified as described earlier in pediatric patients undergoing CCVR, the NIVA value correlates moderately with blood loss and fluid administration and is superior to MAP, indicating a potential use of NIVA for goal-directed fluid therapy in the pediatric perioperative arena. The strong concordance between the directional change in NIVA value and change in net volume (87.5%) utilizing the current algorithm suggests that the underlying morphological waveform changes are similar and inherent in both children and adults.

There are several limitations to this study. First, the sample size was small. However, this was a pilot study aimed toward demonstrating proof-of-concept of this novel technique in children, and we were successfully able to demonstrate a significant correlation even with relatively few subjects. Second, only a single surgical procedure was chosen for enrollment. CCVR was felt to be ideal for this pilot study to analyze rapid changes in volume status due to a high rate and extent of blood loss and need for resuscitation compared to other types of pediatric surgeries. Future clinical trials will be aimed at comparing NIVA to additional objective parameters including calculated blood loss, gravimetric blood loss, MAP, heart rate, systolic blood pressure, urine output, plethysmography variability index, pulse pressure variation, and serum lactate, during various types of pediatric surgeries. Additionally, we plan to assess more granular changes in NIVA signal during times of rapid blood loss or resuscitation and evaluate the efficacy of NIVA in optimizing intraoperative fluid and blood management.

## Conclusions

This study suggests that NIVA is a safe, reliable, non-invasive, quantitative method of measuring intravascular volume status in pediatric patients undergoing high-risk surgery, with the potential to guide goal-directed fluid therapy. This preliminary study also suggests that NIVA correlates more closely to real-time intravascular volume changes than MAP in children, and these observed trends justify further investigation.

## Supporting information

S1 Data (XLSX)
